# Embolization of Left Ventricular Outflow Tract Calcification After TAVR With a Self-Expanding Valve

**DOI:** 10.1016/j.jaccas.2025.105064

**Published:** 2025-09-24

**Authors:** Tetsuma Oyama, Hirohisa Endo, Atsumi Oishi, Shoichiro Yatsu, Tomohiro Kaneko, Hideki Wada, Manabu Ogita, Kan Kajimoto, Tohru Minamino, Minoru Tabata

**Affiliations:** a Department of Cardiovascular Surgery, Juntendo University Shizuoka Hospital, Izunokuni, Japan; b Department of Cardiovascular Medicine, Juntendo University Shizuoka Hospital, Izunokuni, Japan; c Department of Cardiovascular Biology and Medicine, Juntendo University Graduate School of Medicine, Bunkyo-ku, Tokyo, Japan; e Japan Agency for Medical Research and Development-Core Research for Evolutionary Medical Science and Technology (AMED-CREST), Japan Agency for Medical Research and Development, Tokyo, Japan; d Department of Cardiovascular Surgery, Juntendo University Graduate School of Medicine, Bunkyo-ku, Tokyo, Japan

**Keywords:** coronary obstruction, distal opening techniques, self-expanding valve

## Abstract

**Background:**

Severe aortic stenosis with extensive left ventricular outflow tract (LVOT) calcification poses challenges during transcatheter aortic valve replacement.

**Case Summary:**

An 85-year-old man with severe aortic stenosis and extensive annular-to-LVOT calcification underwent transcatheter aortic valve replacement with a Navitor system (Abbott). To avoid conduction disturbances, the valve was deployed within the membranous septum under intracardiac echocardiography guidance, using partial recapture and the distal opening technique. Immediately after the procedure, displaced LVOT calcification embolized the left main coronary artery, leading to cardiogenic shock. The patient was successfully treated with emergency percutaneous intervention and was discharged without other embolic events.

**Discussion:**

The LVOT calcification, which showed no mobility preoperatively, appeared mobile after the procedure, suggesting that device manipulation may have caused its disruption and embolization.

**Take-Home Message:**

It is important to be aware of the possibility that a partially retracted, slightly opened self-expanding valve may interact with LVOT calcification.

## Graphical Abstract

**Figure:**
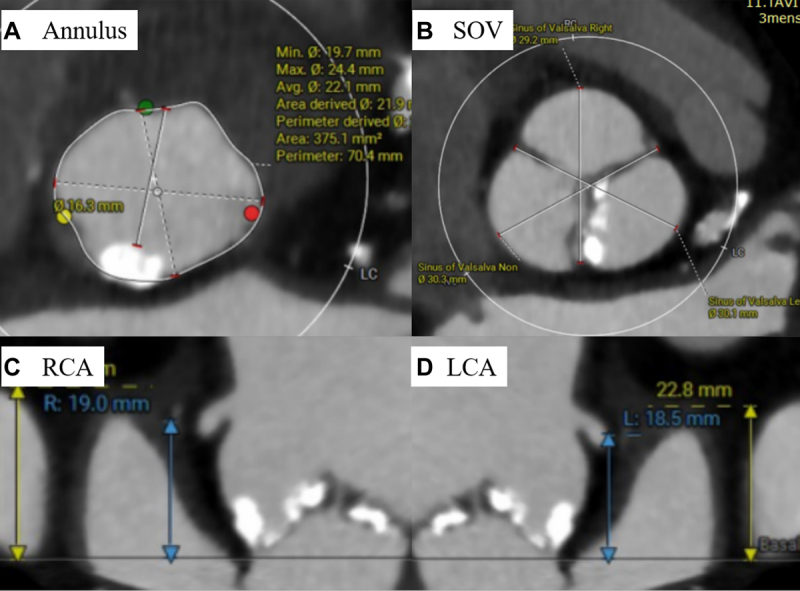

## History of Presentation

An 85-year-old man presented to his primary care physician after having experienced exertional dyspnea for approximately 1 year. A grade 3/6 ejection systolic murmur was noted, and he was referred to us for further evaluation and treatment. Severe aortic stenosis was diagnosed on transthoracic echocardiography (TTE). Despite his age, he could walk without a cane, demonstrating functional independence. He also had chronic obstructive pulmonary disease (COPD), which led to an assessment of high surgical risk. Consequently, we decided to undergo transcatheter aortic valve replacement (TAVR).

**Take-Home Messages**

• We performed TAVR using a Navitor valve in a patient with extensive calcification protruding into the LVOT using partial recapture and the distal opening technique.
• It is important to be aware of the possibility that a self-expanding valve that is partially retracted into the sheath and slightly opened may interfere with calcification in the LVOT.

## Past Medical History

The patient was undergoing inhalation therapy for COPD and had a history of chronic kidney disease.

## Differential Diagnosis

Coronary obstruction generally occurs when the native leaflet occludes the coronary ostium, or when bulky leaflet calcification becomes sequestered in a small sinus of Valsalva. Aortic dissection can also be a cause. Additionally, air, thrombus, or fragments from the aortic valve complex may be dislodged during a procedure and embolize to the coronary arteries.

## Investigations

**Figure 1:**
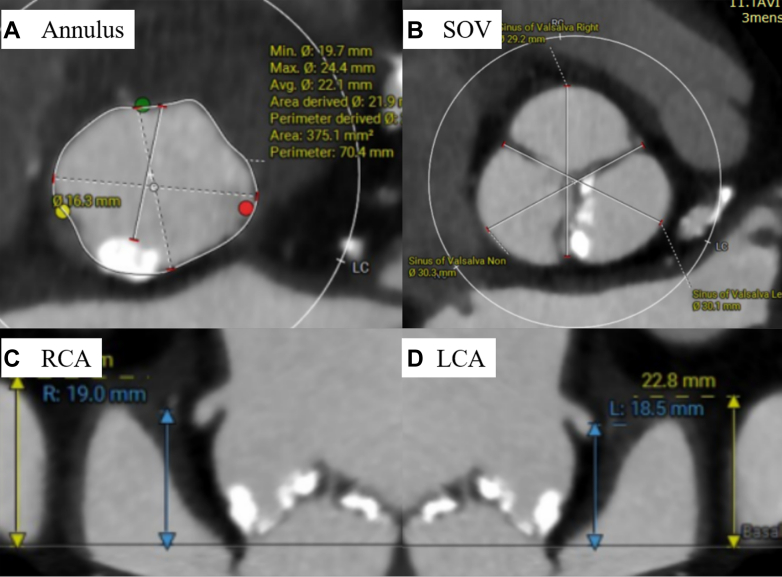
Preprocedural Computed Tomography Images of the Aortic Valve Complex Shown are images of the (A) annulus, (B) sinus of Valsalva (SOV), (C) right coronary artery (RCA), and (D) left coronary artery (LCA).

**Figure 2:**
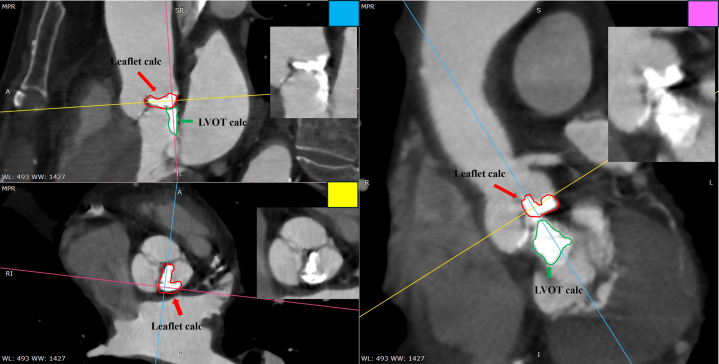
Preprocedural Computed Tomography Showing Annular-to-LVOT Calcification Preprocedural multiplanar reconstructed computed tomography images demonstrate severe calcification extending from the annulus to the LVOT. The pink, yellow, and blue lines indicate the reconstructed planes. Red arrows mark leaflet calcification, and green arrows indicate extensive LVOT calcification. LVOT = left ventricular outflow tract.

**Figure 3:**
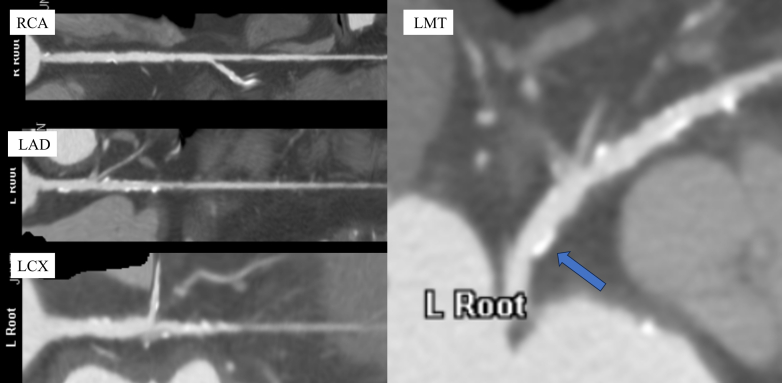
Preprocedural Coronary Computed Tomography Angiography Mild calcification is noted in the left main trunk (arrow), with no stenosis observed. LAD = left anterior descending artery; LCX = left circumflex artery; LMT = left main trunk; RCA = right coronary artery.

**Figure 4:**
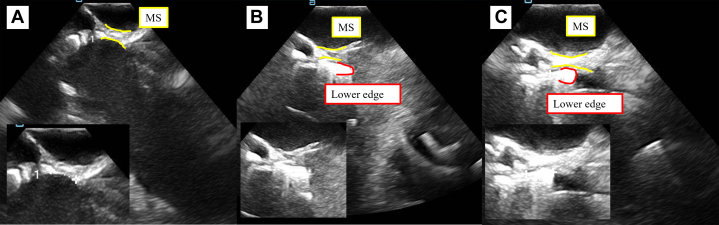
Transjugular Intracardiac Echocardiograms of Valve Deployment (A) Predeployment intracardiac echocardiogram showing the membranous septum (MS) length measured at 4.7 mm, which was consistent with the preprocedural computed tomography measurement. (B) First valve deployment attempt demonstrating the valve's lower edge extending beyond the MS and reaching the muscular septum. (C) Final deployment position showing the valve's lower edge fixed at a depth of 4.0 mm from the annulus, without extending beyond the MS.

Preoperative electrocardiogram showed sinus rhythm, heart rate of 56 beats/min, first-degree atrioventricular block, and left anterior fascicular block. TTE revealed a left ventricular ejection fraction of 64%, aortic valve peak flow velocity of 4.3 m/s, mean aortic valve pressure gradient of 43.8 mm Hg, and an aortic valve area of 0.79 cm^2^. The anatomy of the aortic valve complex as assessed by contrast-enhanced computed tomography was as follows: annulus perimeter of 70.4 mm, annulus area of 375 mm^2^, sinotubular junction of 26.1 mm, sinus of Valsalva (left coronary/right coronary/noncoronary) of 30.1/29.2/30.3 mm, with coronary heights (left/right) of 18.5/19.0 mm, and membranous septum length of 5.0 mm (Figures 1A to 1D). Extensive calcification was present on the non–coronary cusp side, extending from the annulus to the left ventricular outflow tract (LVOT) (Figure 2). The ostial right coronary artery and the middle portion of the left anterior descending artery (LAD) showed 50% narrowing, while calcification was present in the left main trunk (LMT), without narrowing (Figure 3). Extensive calcification from the annulus to the LVOT indicated a higher risk of annular rupture with a balloon-expandable valve. Thus, a self-expanding valve (SEV) was considered more suitable; the Evolut FX (Medtronic) and Navitor Vision (Abbott) were available in Japan at the time of the procedure. Considering the potential reduction in paravalvular leak with the NaviSeal, the Navitor valve was deemed a better choice. Based on these findings and to avoid pacemaker implantation, our heart team planned TAVR using a 25-mm Navitor Vision under local anesthesia via the transfemoral approach with intracardiac echocardiographic guidance (Figure 4A).

## Management

### Primary management

The short diameter of the annulus was approximately 20 mm; however, owing to calcification protruding inward, predilation was performed using a 16-mm Z-MED balloon (NuMed), achieving satisfactory expansion. Subsequently, a 25-mm Navitor valve was advanced into the aortic valve using the cusp-overlap technique and was deployed up to the point of no recapture. After verifying the device placement depth via intracardiac echocardiography and aortography, the initial placement was deemed too deep (Figure 4B), and a partial recapture was performed on the second attempt. Unfortunately, this resulted in a high implantation, increasing the risk of pop-up; consequently, 2 recaptures were necessary using the distal opening technique (Video 1). The valve was positioned satisfactorily without any conduction disturbances (Figure 4C). However, the mean aortic valve pressure gradient remained moderately stenotic at 25 mm Hg (preprocedural: 64 mm Hg). There was no nonuniform expansion phenomenon, and postdilation with the previously used 16-mm Z-MED balloon did not achieve a satisfactory reduction in the pressure gradient. Considering further dilation with a larger balloon, we noticed calcification under the valve with mobility on TTE and intracardiac echocardiography (Videos 2 and 3), which could risk embolization with additional balloon interference. The patient was hemodynamically stable, exhibited no electrocardiogram changes, and had comorbid COPD; therefore, we judged that the benefit of converting from local to general anesthesia to perform transesophageal echocardiography (TEE) was limited. We therefore decided to conclude the procedure and return the patient to the intensive care unit. The patient experienced bradycardia, hypotension, and altered consciousness immediately after returning to the intensive care unit. Electrocardiogram revealed ST-segment elevation in leads I, aVL, and V2 to V6. TTE revealed akinesis in the anterolateral wall, leading us to suspect coronary occlusion.

### Secondary management

**Figure 5:**
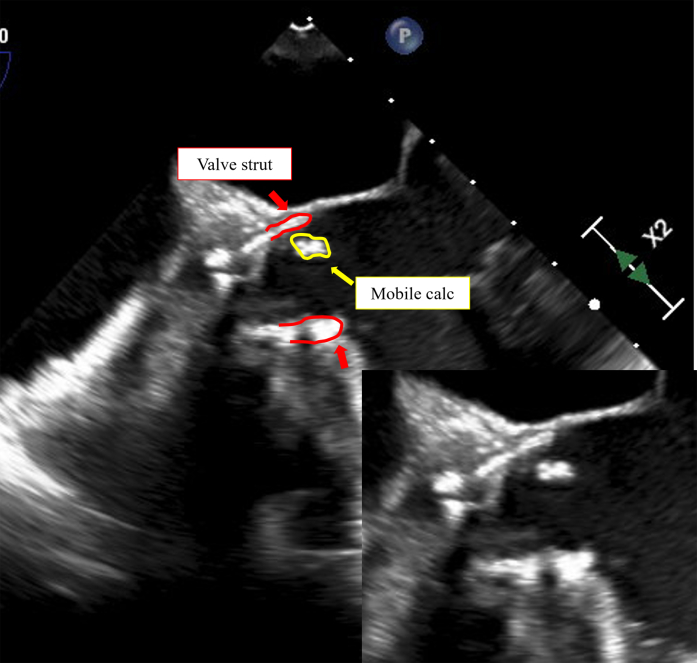
Transesophageal Echocardiogram During Percutaneous Coronary Intervention The area outlined in red indicates the valve strut, and the area outlined in yellow shows the mobile calcification located inside the strut. This is further demonstrated in Video 4.

**Figure 6:**
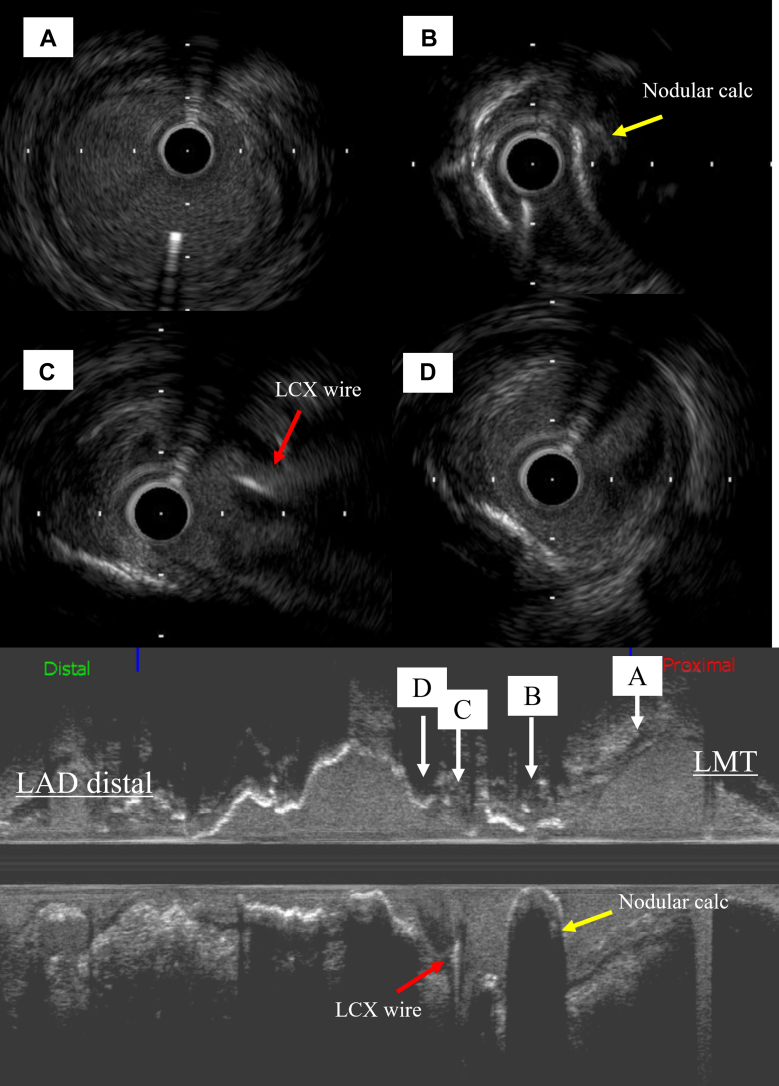
Intravascular Ultrasound Images (A) Ostial left coronary artery. (B) An eccentric calcified lesion with acoustic shadowing. (C) Bifurcation of the distal LMT to the circumflex artery. Mild atherosclerotic plaque is noted from the 9 o'clock to 1 o'clock direction. (D) Just proximal of the LAD. calc = calcification; LAD = left anterior descending artery; LCX = left circumflex artery; LMT = left main trunk.

We promptly transported the patient to the catheterization laboratory and established venoarterial extracorporeal membrane oxygenation. TEE revealed a mobile, calcified mass at the lower and inner edges of the valve (Figure 5, Video 4). Coronary angiography revealed a radiolucent area of the LMT (Video 5). A guide catheter (Judkins left 3.5, 6-F, Mach 1) was easily inserted into the left coronary artery. Initially, we attempted to cross the lesion using a soft wire (SION blue, Asahi Intecc); however, the lesion was rigid and impassable. Therefore, we successfully passed a tapered wire (XT-A, Asahi Intecc) with a microcatheter (Corsair, Asahi Intecc) through the LMT-LAD. The lesion was dilated with a 2.5-mm balloon (Ryurei, Terumo), achieving TIMI flow grade 3 reperfusion. Intravascular ultrasound showed that the lesion in the LMT was eccentrically calcified with acoustic shadowing, resembling a calcified nodule (Figure 6). Preoperative computed tomography showed calcification in the LMT but no severe stenotic lesions. Some LVOT calcifications likely interfered with the valve and were dislodged. The aspiration device was ineffective, and a 3.5 × 26 mm drug-eluting stent (Onyx Frontier, Medtronic) was placed. Immediately after recanalization, TEE showed clear recovery of left ventricular contractility, and hemodynamics stabilized, allowing removal of extracorporeal support in the catheterization laboratory and subsequent return to the intensive care unit. On the following day, the patient was weaned off the ventilator without any neurological defects. No signs of embolic events were noted in the limbs or visceral arteries, and the patient was discharged on foot on day 12 of hospitalization.

## Outcome and Follow-Up

**Figure 7:**
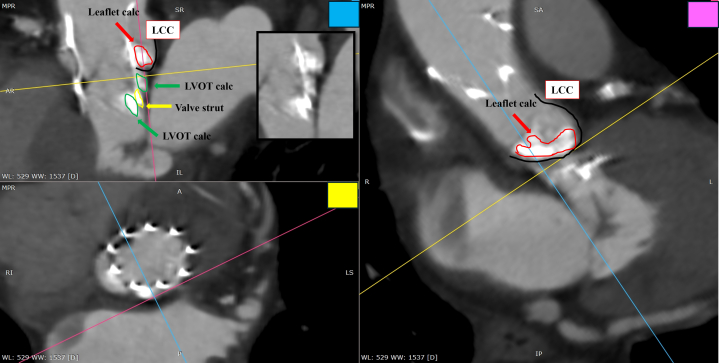
Postprocedural Multiplanar Reconstructed Computed Tomography The red arrows indicate leaflet calcification, the green arrows indicate LVOT calcification, and the yellow arrow indicates the strut at the valve's lower edge. calc = calcification; LCC = left coronary cusp; LVOT = left ventricular outflow tract.

Postprocedural multiplanar reconstructed computed tomography images showed calcification below the left coronary cusp divided into components inside and outside the strut, with the lower edge of the valve projecting into the LVOT (Figure 7). One month after discharge, the patient was in good health and visited the hospital for follow-up visits. TTE revealed calcification at the lower end of the valve; however, no change in size or mobility was noted, nor were there any signs of heart failure or new embolic events. The patient's symptoms improved. At the 1- and 4-month follow-up visits, the mean gradient was <10 mm Hg, with trace paravalvular leak. Given the patient's advanced age and surgical risk, we chose conservative observation.

## Discussion

Coronary obstruction after TAVR is rare but potentially life threatening, with a recent report indicating an occurrence rate of 0.8%.^1^ Risk factors include low coronary height, small valve complex, long leaflets, severe calcification, and high implantation.^2^ As reported from Japan, acceleration of flow in the LMT detected by TEE may allow early identification of coronary artery obstruction, even without hemodynamic instability.^3^ However, in this case, embolism was the main pathology, and TEE may not have enabled early detection of the complication.

SEVs allow repositioning via recapture, with partial recapture enabling small adjustments.^4^ The softer shaft of the Navitor can make coaxiality harder to maintain, increasing the risk of deep placement, pop-up, and conduction disturbance. The distal opening technique helps achieve proper placement,^5^ and it was used here. Ideally, the valve is fully resheathed, reopened at 45° to 60°, then pulled up. However, during partial recapture, the “pushing in” action might have caused the lower end of the valve to impale against significant LVOT calcification. There are no reports of similar complications, suggesting the need to consider the possibility of an incompletely opened device interacting with significant calcification in the LVOT. Although the association between recapture and embolic stroke varies among studies,^4^^,^^6^ we believe that in cases with extensive LVOT calcification such as this, device manipulation should be minimized.

In this case, whether the calcification originated from the LVOT or the leaflets is important. A reduction in volume could indicate embolization. However, leaflet calcification is compressed in the sinus of Valsalva after implantation, while LVOT calcification is fragmented by the device, making volume comparison difficult. Thus, we cannot conclude that LVOT calcification embolization caused the obstruction. However, based on several circumstantial findings, we hypothesize a possible association: 1) the LVOT calcification appeared to be mobile after valve implantation; 2) the calcific material observed in the sinus of Valsalva was localized near the bottom and not around the ostial LMT (Video 6); and 3) postoperative computed tomography demonstrated fragmentation of the LVOT calcification (Figure 7).

## Conclusions

We present a rare case of LVOT calcification embolized in the coronary artery after TAVR. Especially when using SEVs, it is crucial to be aware that manipulating a valve partially retracted into the sheath may pose a risk of injuring LVOT calcification.

## Funding Support and Author Disclosures

The authors have reported that they have no relationships relevant to the contents of this paper to disclose.

## Appendix

Video 1 Recapture and Redeployment of the Navitor Valve Shown is part of the valve deployment procedure. The valve is being advanced toward the LVOT while still in a partial recapture state.

Video 2 Mobility of LVOT Calcification After Valve Deployment (Transthoracic Echocardiography) Transthoracic echocardiography showing mobile calcification at the lower edge of the valve.

Video 3 Mobility of LVOT Calcification After Valve Deployment (Intracardiac Echocardiography) Intracardiac echocardiography demonstrating mobile calcification at the lower edge of the valve.

Video 4 Mobility of LVOT Calcification After Valve Deployment (Transesophageal Echocardiography) Transesophageal echocardiography demonstrating mobile calcification at the lower edge of the valve.

Video 5 Emergency Percutaneous Coronary Intervention Shown is a radiolucent area in the left main trunk consistent with sub–total occlusion. The valve leaflet stored within the sinus of Valsalva also appears radiolucent but does not reach the area near the left coronary ostium.

Video 6 Comparison of Pre- and Postprocedural Computed Tomography Images The upper part shows preprocedural computed tomography images, while the lower part displays postprocedural images. No embolic material or calcification is observed around the ostium of the left main trunk.
